# Potential of Volunteering in Formal and Informal Medical Education—A Theory-Driven Cross-Sectional Study with Example of the COVID-19 Pandemic

**DOI:** 10.3390/ijerph192416955

**Published:** 2022-12-16

**Authors:** Magdalena Cerbin-Koczorowska, Piotr Przymuszała, Michał Kłos, Dominika Bazan, Paweł Żebryk, Paweł Uruski, Ryszard Marciniak

**Affiliations:** 1 Department of Medical Education, Poznan University of Medical Sciences, 60-806 Poznan, Poland; 2 Students’ Scientific Club of Medical Education, Poznan University of Medical Sciences, 60-806 Poznan, Poland; 3 Promotion and Careers Office, Poznan University of Medical Sciences, 61-701 Poznan, Poland; 4 Department of Hypertensiology, Angiology and Internal Medicine, Poznan University of Medical Sciences, 61-848 Poznan, Poland

**Keywords:** volunteering, community-based education, service learning, medical students, healthcare students, COVID-19 pandemic, professional identity formation, interprofessional education

## Abstract

Students’ volunteering is an effective way to manage health crises, including pandemics. Due to the limited capacity of the healthcare system at the time of the COVID-19 outbreak, the engagement of students in volunteering services seemed invaluable. Based on different teaching–learning theories, in this survey study, we aimed to evaluate the potential of the volunteering service project launched by the Poznan University of Medical Sciences during the COVID-19 pandemic as a learning opportunity for undergraduate healthcare students. The results indicate the potential of involving students in volunteering activities for educational purposes, as well as other values, including attitudes and professional identity development, which could be difficult to realize using traditional teaching methods. However, stimulating students’ reflectiveness seems necessary to reach its full educational effectiveness. Medical teachers should provide students with more opportunities for volunteering and service learning and consider making these a constant element of the curriculum beyond the COVID-19 pandemic.

## 1. Introduction

A growing body of literature recognizes the importance of practice-based learning in the didactic process in medical education. Practice-based learning as an educational method is difficult to define, since this concept contains many learning strategies and is based on various theoretical foundations. However, according to Sheehan and Higgs [1], one of the primary concerns of practice-based learning is “learning for and learning in practice”, which is supposed to prepare individuals for future occupations by engaging students in profession-specific activities performed in a particular workplace, prepared classes, and simulations, where they can deal with practical problems. In contrast to teacher-centered classroom-based learning, in which the main purpose of learning is to transfer theoretical knowledge from teacher to student, the idea of practice-based learning assumes experiential learning situated in the reality of practice, both with other practitioners and professionals [1,2].

Meanwhile, the unprecedented threat to global public health caused by the outbreak of the COVID-19 pandemic caused the worldwide introduction of social distancing and a lack of interhuman contact, including within the higher education sector. As a result, many educational institutions, including medical universities, were forced to switch from traditional face-to-face classes to an online education model. For this purpose, they used and adopted different, already existing strategies, such as video conferencing or social media [3]. However, in the case of medical education, online learning may not necessarily be an optimal solution [4,5]. Despite its many advantages, great potential, and the variety of available e-learning tools, the limitations of online learning should also be considered, including difficulties in learning practical skills, limited contact between teachers and students, and the risk of lowering the quality of learning and assessment [6]. There is also a risk of failing to meet the basic principles of constructive alignment, which assume the need for consistency between learning outcomes and learning activities [7] as a result of the incorrect implementation of online learning in areas such as practical and clinical reasoning or communication skills learning.

It could seem, therefore, that the COVID-19 pandemic constituted a risk for the implementation of practice-based learning in medical education. The limiting of students’ participation in clinical classes impedes achievements of learning outcomes in the domains of practical skills and attitudes. However, at the same time, we have observed a growing engagement of medical students in voluntary services. Different authors have reported on the broad extent of students’ involvement in the fight against the pandemic and various tasks entrusted to them [8,9,10,11,12,13,14]. Moreover, in some countries, decision-makers even created legal incentives for medical students to participate actively in helping the medical staff during the pandemic. In Poland, students who engaged in voluntary work in hospitals and other medical institutions could credit the clinical rotation they missed because of the pandemic, whereas the Italian government decided to graduate final-year students earlier than scheduled, so about 10,000 graduates could legally work with patients in clinics and retirement homes [15]. A similar situation was present in the US, where 13 medical schools also provided early graduation [16]. The decision to include students in the workforce to combat COVID-19 is not a new concept and was facilitated by previous experiences with pandemics and other disasters [16]. Meanwhile, in the study by Rosychuk et al. [17], nearly 95% of respondents (students and staff) declared a willingness to volunteer in the case of a future pandemic influenza. Other studies are also optimistic in this regard. In a study conducted in Peru, 77% of investigated medical students answered that participation in volunteerism during the influenza outbreak is a moral and professional obligation [18]. Similarly, more than two-thirds of respondents to Yonge et al. [19] expressed the likelihood of volunteering during a pandemic and regarded it as a professional obligation. According to Holdsworth [20], “student volunteering is increasingly endorsed as a panacea for a broad spectrum of social problems and issues”, which seems promising considering the deficiency in medical staff in different countries. Therefore, this study aimed to investigate the potential of using volunteer work in educating future adepts of medical professions and placing volunteering in well-established teaching–learning theories.

## 2. Materials and Methods

### 2.1. Study Setting and Design

**Figure 1:**
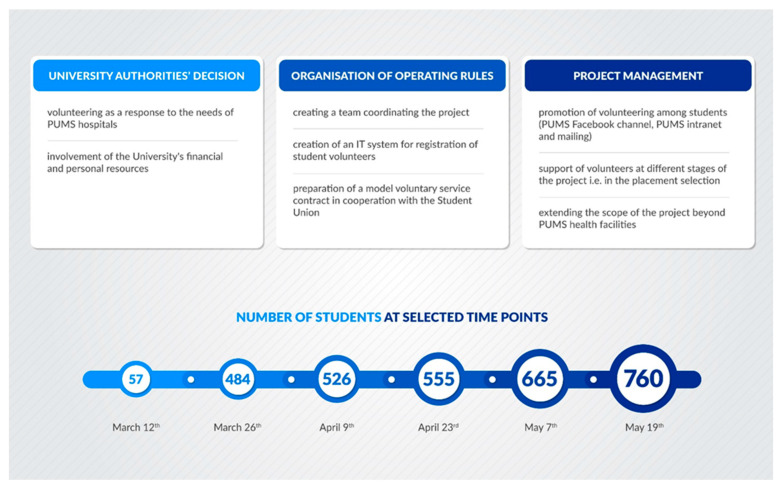
Stages of the development and implementation of the volunteering service project.

On 12 March 2020, a student-volunteering service project was launched by the Poznan University of Medical Sciences (PUMS) Rector as a response to the needs of PUMS medical facilities against the COVID-19 outbreak. In the following weeks, the scope of volunteering expanded to include further units and initiatives. Figure 1 presents the stages of volunteering organization and its growing popularity among students. As of 19 May 2020, students volunteered in 28 units, including hospitals, hospital pharmacies, outpatient clinics, primary care, the local Sanitary and Epidemiological Station, a laboratory for COVID-19 testing, a mobile swab collection unit, a quarantine unit, as well as a call center.

Of the 6308 PUMS students in undergraduate programs, 760 students applied during the first ten weeks of the project, representing 12% of the student population. The majority (71%) were female, and 66% were medical students. Within 16 out of 28 units, student volunteers representing at least two different professions worked together. Table 1 shows the involvement of students in the work of individual institutions and the scope of the tasks they performed.

The survey used in this study contained closed-ended and open-ended questions and was drafted by the research team at the Department of Medical Education. The intent behind the survey was to make it relatively short in order not to discourage students from participation but, at the same time, to cover teaching and learning theories and concepts relevant to volunteering, such as learning outcomes, professional identity formation, interprofessional education, and reflective learning, as well as sources of students’ support and motivation. Additionally, the survey contained questions on participants’ demographic data such as their gender, study field, and place of volunteering. Subsequently, a draft of the survey was piloted among ten student volunteers, whose feedback contributed to the final version of the survey used in the study.

The survey was distributed online among potential participants between January and March 2021. The online questionnaire was promoted in social media, including among the group of student volunteers from PUMS engaged in volunteering service during the COVID-19 outbreak. The decision to end the data collection process was made 4 weeks after receiving the last completed response. The quantitative data obtained during the study were analyzed with the Statistica software (StatSoft) (version 13.3) (TIBCO Software Inc., Palo Alto, CA, USA) using the Mann–Whitney U and Wilcoxon signed-rank tests. Qualitative data from open-ended questions were content analyzed by two independent researchers.

### 2.2. Study Group

The link with the survey was opened by 202 potential participants. However, only 70 of them started providing answers, giving a response rate of 34.7%. Among them were 52 females, 17 males, and one student who chose not to reveal their gender. They represented different study fields offered at PUMS, including medicine (*n* = 42), nursing (*n* = 7), laboratory diagnostics (*n* = 6), medical rescue (*n* = 5), pharmacy (*n* = 4), dentistry (*n* =3), and midwifery (*n* = 2). The average time of their participation in volunteering was 8.8 weeks. Participants’ volunteering settings involved: hospital pre-triage (*n* = 18), hospital wards (*n* = 10), primary healthcare clinics (*n* = 10), hospital emergency departments (*n* = 9), and the University Coronavirus Laboratory (*n* = 9), among others. Given that some participants did not provide answers to all questions, for clarity of interpretation, the results of the study are presented below with the number of students that answered each question.

### 2.3. Ethical Issues

The study project was presented to the Bioethical Committee of the Poznan University of Medical Sciences, which decided that given the survey nature of the study, it did not require the Committee’s approval under Polish law. Nevertheless, we took efforts to ensure its highest ethical standards. The survey was anonymous and completely voluntary to the participants. In the survey description, they were also informed about the aims, procedure, and course of the study, and they were asked to provide consent for participation before starting the survey.

## 3. Results

**Figure 2:**
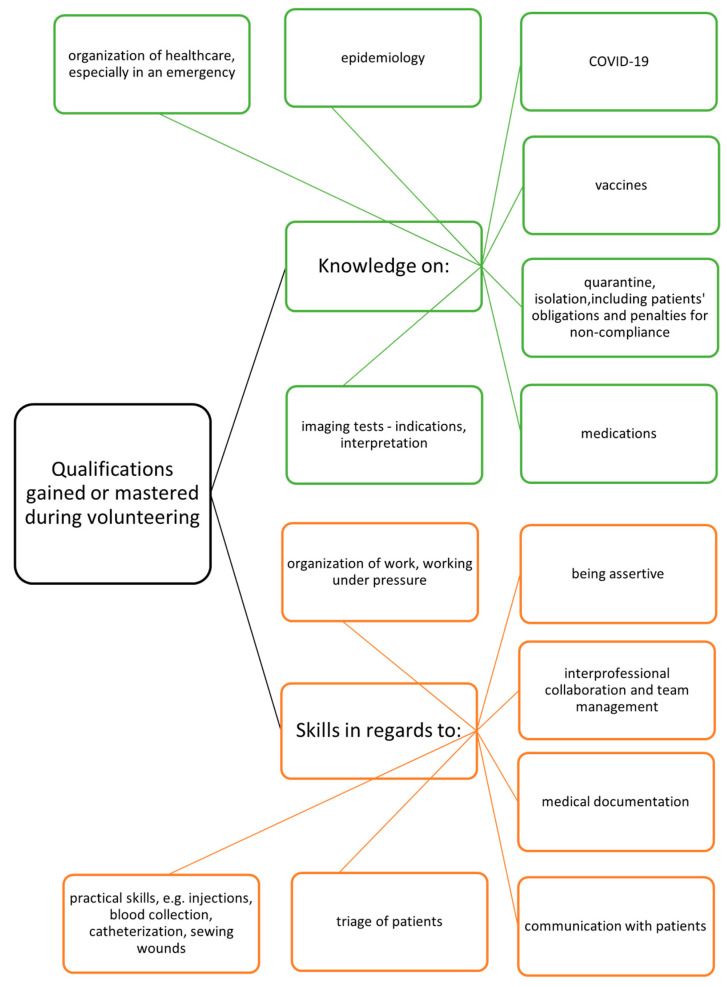
Knowledge and skills acquired or mastered by students during volunteering as reported by students.

Most students believed that their participation in the volunteering activities allowed them to gain new knowledge and skills apart from those during their studies (50.7% and 52.4%, respectively), and only 31.9% of them agreed that they used qualifications obtained during their studies (Table 2). The differences between their belief in using previously acquired qualifications and gaining new ones as a result of the experience were statistically significant both for knowledge (*p* = 0.008) and skills (*p* = 0.008). Students also provided examples of newly acquired knowledge and skills, which were categorized and presented in Figure 2.

Participation in the volunteering experience also seemed to influence the professional identity of respondents. For 53.2% of them, it influenced their way of seeing themselves in their future professional role, and 41.0% were confirmed in their conviction of wanting to practice a medical profession in the future. As a result of the volunteering activity, 63.8% of students identified more strongly with representatives of healthcare professions in general and 46.6% with representatives of their future profession (Table 2).

In terms of interprofessional relations, 78.9% of students had the opportunity to collaborate with representatives of other medical professions, and 63.8% had the opportunity to collaborate with students of other faculties, which was significantly less than the former (*p* = 0.031). Though most students (53.4%) believed that representatives of every profession had the same goal during their voluntary work, only 32.8% believed that representatives of each profession had the same status in the team during their voluntary work (Table 2).

Members of the medical personnel and other students were, in respondents’ opinion, the most common source of support during volunteering, whereas local government representatives and University authorities were its least common source (Table 3). The differences between the perceived level of received support depending on its source were mostly statistically significant, as presented in Table 4. Most students (86.0%) agreed or strongly agreed that they had occasion to discuss the volunteering experiences with their peers, whereas only 35.1% discussed it with medical personnel and 12.3% with academic teachers. The differences in their opinions in this regard were also statistically significant, as presented in Table 4. Additionally, male students statistically believed more strongly that they had occasion to discuss their experiences with team members (*p* = 0.046). No other significant differences were noticed.

The most commonly mentioned factors that influenced students’ decision to participate in volunteering were the possibility of passing their holiday internships, opportunities for personal development and acquiring new knowledge and skills, their interest in medicine, altruistic factors and willingness to help those in need, and curiosity. On the other hand, the least important factors included the feeling of expectations from the academic community, religious or philosophical factors, the possibility of collaboration with students of other faculties, the possibility of cooperation with representatives of other medical professions, and the feeling of such expectations from family, friends, and acquaintances (Table 5). No statistically significant differences were noticed in students’ motivation, except for the possibility of collaboration with students of other faculties, which was significantly more frequently indicated by female students (*p* = 0.034).

## 4. Discussion

Given the enormous challenge of continuing the education process during the COVID-19 pandemic crisis, the scientific world has intensified its work to assess the consequences of its various aspects on the quality of the education provided. Some authors have indicated the effect of the pandemic on the rate of employment as the main concern coming to mind [21], and others have noticed the need for teachers and students to be more oriented toward different online education tools as a lesson from the COVID-19 pandemic [22]. However, medical education should be viewed within its specificity. The rate of employment not only has not declined, but the demand for medics increased during the pandemic. At the same time, the pandemic coincided in many countries with staff shortages that already existed beforehand. For example, in Poland, in 2017, the number of physicians per 100,000 inhabitants was equal to 238, which was the lowest in the European Union [23]. Further, the number of nurses was lower than in most European countries (510 per 100,000 inhabitants) [24]. Staff shortages seem to be common also in other, even more prosperous countries. For example, in the United States, the number of job offers for nurses was estimated to be higher than for any other occupation by 2022 [25]. Though medical universities have implemented online solutions [6], this education form does not enable students to acquire all the qualifications necessary to practice their profession. At the same time, as mentioned above, we observed that medical universities became strongly involved in the fight against the pandemic at the institutional level, enabling screening, creating temporary hospitals, and organizing volunteer support. Maintaining high-quality medical education with limited access to patients was challenging. Even simulation methods cannot replace the practice gained in the workplace. Considering all of the above, this study aimed to examine the potential of using volunteer work in educating the adepts of the medical professions and to place volunteerism into well-established teaching–learning theories.

### 4.1. Outcome-Based Education

The pillar of modern education is the student-centered approach. When designing healthcare curricula today, a special emphasis is put not so much on the teaching process but on the educational product. Nowadays, a key element of designing the educational process is defining the educational goals and specific, measurable learning outcomes to which, according to constructive alignment assumptions, the successive stages of educational intervention design are adjusted [26,27]. Students, teachers, and decisionmakers should assume that the common goal is students’ achievement of specific qualifications. However, the paths to achieving this may vary. One of them may be volunteering activities. In our study, more than half of the students agreed or strongly agreed that the volunteering experience allowed them to gain new knowledge and skills. Moreover, they provided many examples of these experiences in response to open questions. In order to assess the potential of using volunteering in the course of formal learning, we additionally analyzed the standards of teaching in medical faculties according to the Ordinance of the Ministry of Science and Higher Education on the standards of education for the faculties of: medicine, dentistry, pharmacy, nursing, midwifery, laboratory diagnostics, physiotherapy, and medical rescue [28]. The learning outcomes were selected taking into account both the scope of tasks entrusted as part of the voluntary service at PUMS and the specificity of tasks entrusted to students as part of volunteering at other universities described in the literature cited above. As presented in Appendix A, in the example of Polish curriculum, if decisionmakers were to take action to describe the tasks carried out by students during voluntary activities with the help of learning outcomes, there would be a potential to integrate these activities into the realization of the regular formal curriculum. In this way, curricula design could assume the opportunity for the realization of selected learning outcomes in the form of volunteering. However, the process should be thoroughly planned and thought through. The chosen learning outcomes should be possible to achieve by students during the given activity with respect to such issues as students’ pre-existing competencies, the tasks entrusted to them, and the time planned for their participation in volunteering. Moreover, not only should the chosen activities match the expected learning outcomes, but according to the abovementioned constructive alignment principle, adequate assessment strategies should also be planned to verify the achievement of the learning outcomes and provide learners with feedback on their performance [26,27].

### 4.2. The Use of Volunteering in Professional Identity Formation

Taking into account the specificity of the tasks performed by students, their participation in volunteering may also contribute to the development of qualifications, the integration of which into established concepts of medical education seems problematic [29]. Expert performance is not only about presenting knowledge and skills but, most of all, the readiness to use them to carry out professional tasks in an everyday, often disorderly environment [30]. However, even that may not be enough. According to Hafferty, “the fundamental uncertainties that underscore clinical decision making and the ambiguities that permeate medical practice require a professional presence that is best grounded in what one is rather than what one does”. This leads to a shift from the emphasis on medical professionals’ actions to their identity, with ‘being’ viewed as a better basis for professional behaviors than ‘doing’ [31]. In this aspect, the role of medical education is to provide students “with a professional identity so that he comes to think, act, and feel like a physician”, which can gradually occur due to their conscious decisions and the impact of their experiences, both clinical and non-clinical. At the same time, professional identity formation should be an active, dynamic, and constructive process crucial to competency-based education [32,33]. It is the responsibility of medical educators to design curricula in order to guide, support, and challenge students through the process [32]. However, formal teaching seems insufficient to ensure this process, and informal contacts are understood to have a bigger potential in developing the professional identities of students. Further, socialization with patients and medical team members is considered a vital source for the construction of professional identity [34]. Importantly, the process does not seem linear, with some events having a progressive and solidifying effect, for instance, contact with death [31]. The volunteering activity and the pandemic situation could also be regarded as such events. Most of our respondents noticed that the volunteering experience influenced their perception of themselves in their future professional roles and increased their identification with representatives of healthcare professions. Admittedly, this effect was less visible for their belief in their willingness to practice the medical profession in the future and their identification with representatives of their own future profession. However, we did not measure their beliefs before their volunteering involvement, but rather focused only on the change as a result of it. It may be possible, therefore, that their base beliefs were already firmer in regard to these questions. Meanwhile, reports from other examples of students’ involvement in community service show its role in connecting the process of acquiring knowledge and professional identity development [34]. Given the significance of interactions with patients, peers, and other team members in complex environments in the process of active professional identity formation, as well as its place in contemporary medical education [32,35], it seems that medical universities should increase the opportunities for students for their professional identity formation [34].

### 4.3. Volunteering as an Opportunity to Build Interprofessional Relationships

According to World Health Organization experts, the only way to effectively manage health crises, such as epidemics and pandemics, is the ability of healthcare forces to stand shoulder-to-shoulder regardless of their profession to secure the health needs of local society [36]. Guidelines on the implementation of effective IPE initiatives [37,38] underlined the importance of hands-on interprofessional practices, which allow students to engage in formal and informal team activities. Working in an interprofessional environment enables students not only to ‘think’ but also to ‘act’ as team members, which provides the opportunity for reflection on the working relationships they have established and the factors that may shape effective teamwork.

Allport [39] points out, however, that the contact between representatives of different professions alone is not sufficient. His intergroup contact theory assumes that four factors are essential to improving intergroup attitudes: (1) the equal status of team members, (2) common goals, (3) intergroup cooperation, and (4) the support of institutional authorities. Volunteering launched at medical colleges to fight the COVID-19 pandemic creates an opportunity to evaluate the intervention in terms of these criteria.

First, in light of the underperforming healthcare system, each volunteer engaged equally in providing patient-centered services, regardless of their professional background. However, according to students’ responses, only 32.8% believed that representatives of each profession had the same status during their voluntary work, which seems worrisome. Previous studies have shown that medical and nursing students view volunteering during a pandemic as their professional obligation [19,40]. Until recently, most interprofessional interventions focused on these two groups of students [41]. Our experience confirmed the commitment of medical students but also showed involvement among students of 14 other health-related majors.

Second, the volunteers shared a common goal—fighting the COVID-19 pandemic. Most students in our study believed that representatives of every profession had the same goal during their voluntary work. The experience of interprofessional collaboration at the time of the Ebola outbreak shows that working toward a common goal in challenging situations fosters partnership and teamwork between different medical professions, as members have a sense their contribution is equally valued [42].

Third, students not only interacted with each other, but by working together on specific tasks, they became dependent on each other. According to the respondents, 78.9% had the opportunity to collaborate with representatives of other medical professions, and 63.8% had the opportunity to collaborate with students of other faculties. Additionally, healthcare team members and other students were also the sources of the most support felt by the respondents. Patient care in pandemic conditions facilitates intergroup cooperation, as it requires attention to the patient’s welfare while mitigating risk for the healthcare team [42]. It is also worth noting that during the recruitment process, each volunteer had the opportunity to choose his placement to best match his qualifications and preference with the assigned team. The importance of the volunteer’s choice aspect is two-fold. On the one hand, building interprofessional relationships is possible when individual members are aware of their responsibilities, and only in a safe environment can they learn the roles of other professionals and, over time, take on some of the shared tasks [38]. On the other hand, using acquired skills in a real-world context increases the effectiveness of educational experiences, and students better understand their role in meeting the health needs of the community [43].

Finally, volunteers received institutional support from the University authorities and external stakeholders, i.e., local representatives of a central government. The PUMS authorities also decided that participation in the voluntary service would count as completing a formal internship that each student is required to take for a month during the summer break. Surprisingly, however, this support did not seem to be enough for the respondents, among whom only 21.1% and 8.8% said they felt this support from the University authorities and local government, respectively. As we did not ask students for justification for their responses, we can only suspect that some forms of support were unrealized or, on the contrary, seemed obvious for students, such personal protection equipment (PPE), coordination of the volunteering activities, a video clip with PUMS authorities expressing gratitude for students’ involvement, or the abovementioned possibility of completing internships. Meanwhile, some of these also required big effort due to, for instance, severe shortages of PPE due to increased demand during the pandemic or the frequently changing guidelines and recommendations impeding the coordination process. At the same time, we also acknowledge that students might have had some other needs and expectations that were not met by the authorities. Further studies are required to examine this phenomenon more thoroughly and uncover students’ expectations in this aspect.

Volunteering seems to allow students to experience interprofessional teamwork and collaboration and to understand the roles and contributions of the professions with which they interact, fulfilling two primary purposes of interprofessional practice education [38]. At the same time, it may provide the opportunity for informal interprofessional learning, strongly recommended by Rees et al. [44], aimed to allow a student to carefully observe other professions at work and build their self-confidence in communicating with members of different disciplines. It can thus be suggested that in the absence of mandatory interprofessional classes at PUMS, volunteering can be considered an opportunity for all healthcare students to work together to safeguard the health needs of the local community, despite the fact that, as our results show, the collaboration with students from other faculties was not a common motivation to volunteer. Noticeably, this was more often indicated by female students, which is consistent with previous reports on the readiness of Polish students for interprofessional learning [45]. The presented model of voluntary service seems to provide a unique opportunity to meet the challenges of healthcare during the pandemic while shifting away from a healthcare delivery model by independent tribes of healthcare professionals towards one in which empathy and solidarity take precedence—a model in which members of all healthcare-related professions work together for patients’ benefit. This seems especially important given the potential multidimensionality of these challenges from the medical staff’s perspective. For example, a recent study by Revythis et al. [46] showed a mix of positive and negative consequences of the COVID-19 pandemic for junior and middle-grade doctors, including its positive financial impact, the increased workload associated with the pandemic, and a high personal achievement with low depersonalization and moderate emotional exhaustion levels. Further studies, however, are needed to gather evidence on the educational value of this project, including its importance in shaping interprofessional skills and attitudes among undergraduate students.

### 4.4. Volunteering as an Example of Service Learning

Another aspect of contemporary medical education is the emphasis on using the community setting in the education process [47]. This corresponds with the importance for medical universities to ensure the health of the population, which is often reflected in their mission statements [48]. This is believed to have many advantages, including enhancing knowledge, skills and attitudes, deepening students’ understanding of health, illness, and the role of social and environmental factors on disease cause and prevention, promoting a patient-oriented perspective, and enhancing interprofessional collaboration, among others [47]. Community-based education also gives a sense of working for the benefit of the community, and it builds commitment [47]. It seems especially useful in the case of complex topics, which could be difficult to convey with traditional teaching methods, including professionalism or cultural competencies [48]. Meanwhile, as studies show, community-based learning allows students to grasp a better understanding of and to gain new insights into these issues [48]. Given the overlapping of the terms and concepts of community-based and service learning [48], in this paper, they will be discussed together. Service learning puts emphasis on students’ active involvement in service to the community combined with subsequent continuing reflection on their experiences with the idea of aiding them in the rediscovery of their initial and noble reasons for pursuing medicine [48]. However, it should also be coherent with the needs and concerns of the community [49]. Further, Quinn et al. [50] notice that these programs should benefit both students and the community. Given the abovementioned staff shortages with a simultaneous increase in the need for healthcare services in the population during the pandemic crisis and students’ gains mentioned in this study, the presented intervention seems to meet this assumption. Although every situation may have educational potential, it is important to remember that we do not necessarily learn from everything we do [51]. The mere placement of a student at a community site for a certain time does not guarantee that the student will achieve the assumed learning outcomes [52]. In order to contemplate and integrate the lessons learned from the students’ community experiences, reflection should take place after such a service-based experience [48]. Aronson [53] defined critical reflection as “the process of analyzing, questioning, and reframing an experience in order to make an assessment of it for the purposes of learning (reflective learning) and/or to improve practice (reflective practice)”. Moreover, the help and support of another person (e.g., a peer, supervisor, or mentor) seems important in realizing the potential of such reflection [30]. However, in the conducted study, it seems worrisome that according to the respondents, only 35.1% had an occasion to discuss their volunteering experiences with the medical personnel and 12.3% with academic teachers. Although 86.0% of the volunteers had an occasion to discuss their experiences with their peers, as our previous research experiences show, at least in Poland, students may place a larger value on feedback from their teachers than that from other students [54]. Therefore, it seems reasonable to recommend that the introduction of volunteering learning should be simultaneously followed by obligatory reflection sessions in the presence of a mentor (e.g., a medical teacher or staff member). Further, a culture feedback, which in Poland is still at a very low level, should be generally promoted among medical students and in the healthcare setting.

### 4.5. Limitations

The presented study has some limitations that should be mentioned. As the data come from only a limited sample of students at a single medical university, they should not be viewed as representative of the population of Polish medical students. Further, given that only some students took the survey, it may be plausible that those who decided to participate in the study could have stronger positions on the topic (both positive and negative) than the rest of the students. Additionally, a disproportion between the number of female and male students can be noted. However, this seems consistent with the gender distribution of students volunteering at our University presented above. Finally, the achieved return rate of 34.7%, amounting to the low number of respondents, limits the confidence in the achieved results and the conclusions drawn from them. Therefore, further studies are needed to support the data from our study on the potential of volunteering in medical education, especially in terms of outcome-based education, professional identity formation, interprofessional education, and service learning. These should be conducted both in different educational settings and countries and on bigger, representative sample sizes of students. Future research could also consider the use of qualitative methodology on students’ experiences and opinions in this regard in order to obtain a larger insight and understanding of the examined phenomena.

## 5. Conclusions

Even with the noticeable interest in aligning the community needs with the medical curricula, much room is still left for the full development of mutual partnership between the community and the faculty members [48]. The use of the outcome-based education approach and the COVID-19 pandemic experiences described in this paper show the potential for rethinking the educational process and using a wide range of learners’ experiences to educate qualified future healthcare professionals. Taking actions by decision- and policymakers to describe the tasks performed by students during voluntary activities with learning outcomes may provide a potential to integrate them into the implementation of the formal regular curriculum. The results of the study show the potential of involving students in voluntary activities, which can serve as a potent tool with the following points in mind:

1. The identified learning outcomes, which could be implemented in the course of volunteering, show that there is a potential for using voluntary activities in the educational process.
2. The participation of healthcare students in volunteering may also have many other values, including building professional identity and attitudes difficult to develop in the course of traditional educational methods.
3. The educational effectiveness of these activities depends on stimulating students’ reflection.
4. This approach can be useful for implementing other volunteer work activities, not only COVID-19 related ones.

## Figures and Tables

**Table 1:** Participation of Poznan University of Medical Sciences student volunteers in various units and the scope of the entrusted tasks (as of 19 May 2020).

Unit	Scope of Tasks	Total Number of Students	Volunteer Structure by Field of Study (n)
Hospitals *	performing initial TRIAGE of patients on admissions interviewing patients in acute care and referring to a doctor in charge assessing the risk of COVID-19 through a questionnaire measuring the temperature of patients and staff entering the hospital assisting in performing lab analyses performing laboratory tests taking and transporting samples scheduling patients for teleconsultation with a doctor and giving short telephone advice providing patients with information on hospital activity during the COVID-19 pandemic period giving advice on how to deal with symptoms indicating COVID-19 infection	422	medicine (300) nursing (41) electroradiology (33) midwifery (17) dentistry (12) laboratory medicine(7) medical rescue (7) dietetics (1) medical biotechnology (1) pharmacy (1) physiotherapy (1) public health (1)
Sanitary and Epidemiological Station (SES)	providing telephone information on the rules of conduct in case of suspected SARS-CoV-2 infection current contact with the authorities conducting epidemiological investigations	36	medicine (18) laboratory medicine (6) pharmacy (4) physiotherapy (3) public health (3) cosmetology (1) medical biotechnology (1)
University Coronavirus Laboratory	reception of samples for testing medical record keeping cooperation with SES and hospitals ordering COVID-19 tests	29	laboratory medicine (22) medicine (5) dentistry (1) midwifery (1)
Drive-through swab collection unit	securing and logistics of personal protection equipment for employees and volunteers sampling and transport of samples medical record keeping patient registration and telephone consultation	28	medicine (17) dentistry (9) dietetics (1) neuroscience (1)
Call-centre for seniors	providing telephone advice on, i.e., rules of conduct in the event of suspected SARS-CoV-2 infection, pharmacotherapy, and occupational therapy	11	pharmacy (5) occupational therapy (4) medicine (2)

* Range of tasks may vary from one hospital to another; SARS-CoV-2—Severe Acute Respiratory Syndrome Coronavirus 2; COVID-19—Coronavirus Disease 2019.

**Table 2:** Student volunteering in terms of qualifications, professional identity, and interprofessional relations.

Statement	Σn	Mean ± SD	M	Q_1_	Q_3_	%n_4+5_
Relationship between the curriculum course and tasks performed during volunteering						
During the voluntary service, I used the qualifications obtained earlier during my studies.	69	2.61 ± 1.45	2	1	4	31.9
Participation in volunteering allowed me to gain additional knowledge apart from that during my studies.	67	3.13 ± 1.66	4	1	5	50.7
Participation in volunteering allowed me to gain additional skills apart from that during my studies.	63	3.16 ± 1.64	4	1	5	52.4
Professional identity						
Participation in volunteering has influenced my perception of myself in my future professional role.	62	3.27 ± 1.55	4	2	5	53.2
Participation in voluntary service strengthened my conviction that I would like to practice a medical profession in the future.	61	3.00 ± 1.48	3	2	4	41.0
Participation in volunteering made me identify more strongly with representatives of the healthcare sector.	58	3.47 ± 1.48	4	2	5	63.8
Participation in volunteering made me more strongly identify with representatives of my future profession.	58	3.12 ± 1.53	3	2	5	46.6
Interprofessional relations						
During the voluntary work, representatives of each profession had the same status in the team.	58	2.83 ± 1.40	3	1	4	32.8
During the voluntary work, representatives of each profession had the same goal.	58	3.41 ± 1.23	4	3	4	53.4
During the voluntary work, I had the opportunity to collaborate with representatives of other medical professions.	57	4.16 ± 1.10	5	4	5	78.9
During the voluntary work, I had the opportunity to collaborate with students of other medical faculties.	58	3.55 ± 1.68	4	2	5	63.8

Σn—total number of respondents; M—median; Q_1_—lower quartile; Q_3_—upper quartile; %n_4+5_—percentage of respondents who selected answers ‘agree’ or ‘strongly agree’.

**Table 3:** Support and reflection opportunities during volunteering.

Statements	Σn	Mean ± SD	M	Q_1_	Q_3_	%n_4+5_
During the voluntary work, I felt the support of:						
the authorities of my university	57	2.23 ± 1.34	2	1	3	21.1
local government authorities	57	1.84 ± 1.16	1	1	3	8.8
academic teachers	57	2.51 ± 1.40	2	1	4	26.3
healthcare team members	57	4.04 ± 1.12	4	3	5	73.7
other students	57	3.93 ± 1.07	4	3	5	70.2
local community	57	2.68 ± 1.42	3	1	4	29.8
I had the opportunity to discuss the experiences gathered during volunteering with:						
academic teachers	57	1.81 ± 1.13	1	1	2	12.3
healthcare team members	57	2.63 ± 1.56	2	1	4	35.1
my peers	57	4.16 ± 1.21	5	4	5	86.0

Σn—total number of respondents; M—median; Q_1_—lower quartile; Q_3_—upper quartile; %n_4+5_—percentage of respondents who selected answers ‘agree’ or ‘strongly agree’.

**Table 4:** Support and reflection opportunities during the volunteering—statistical analysis and *p*-values.

During the Voluntary Work, I Felt the Support of:									
	Authorities of My University	Local Government Authorities	Academic Teachers	Healthcare Team Members	Other Students	Local Community	Academic Teachers	Healthcare Team Members	My Peers
authorities of my university	-	*p* = 0.036	ns	*p* < 0.001	*p* < 0.001	ns			
local government authorities	*p* = 0.036	-	*p* = 0.002	*p* < 0.001	*p* < 0.001	*p* < 0.001			
academic teachers	ns	*p* = 0.002	-	*p* < 0.001	*p* < 0.001	ns			
healthcare team members	*p* < 0.001	*p* < 0.001	*p* < 0.001	-	ns	*p* < 0.001			
other students	*p* < 0.001	*p* < 0.001	*p* < 0.001	ns	-	*p* < 0.001			
local community	ns	*p* < 0.001	ns	*p* < 0.001	*p* < 0.001	-			
I had the opportunity to discuss the experiences gathered during volunteering with:									
academic teachers							-	*p* < 0.001	*p* < 0.001
healthcare team members							*p* < 0.001	-	*p* < 0.001
my peers							*p* < 0.001	*p* < 0.001	-

ns—no statistically significant differences.

**Table 5:** The impact of individual factors on the motivation to participate in volunteering.

Motivation Factors for Volunteering	Σn	Mean ± SD	M	Q_1_	Q_3_	%n_4+5_
the possibility of passing the holiday internships	48	3.81 ± 1.53	4	3	5	72.9
opportunities for personal development, acquiring new knowledge and skills	48	3.42 ± 1.51	4	2	5	62.5
interest in medicine	48	3.35 ± 1.51	4	2	5	56.3
altruistic factors and willingness to help those in need	48	3.40 ± 1.40	4	2	5	54.2
curiosity	48	3.38 ± 1.65	4	1.5	5	54.2
the feeling of being needed by someone	48	3.17 ± 1.45	3	2	4	47.9
filling time and boredom during the lockdown	48	2.92 ± 1.70	3	1	4.5	47.9
sense of pride and satisfaction in helping those in need	48	3.04 ± 1.43	3	2	4	45.8
the possibility of being part of a medical team	48	2.98 ± 1.51	3	1	4	41.7
sense of duty towards the local community	48	2.83 ± 1.64	2	1	5	39.6
feeling that the academic community expects it from me	48	2.56 ± 1.56	2	1	4	35.4
religious or ideological factors	48	2.29 ± 1.49	1.5	1	4	27.1
the possibility of collaboration with students of other medical faculties	48	2.35 ± 1.49	2	1	3.5	25.0
the possibility of collaboration with representatives of other medical professions	48	2.33 ± 1.29	2	1	3	18.8
feeling that family, friends, acquaintances expect it from me	48	1.75 ± 1.12	1	1	2	8.3

Σn—total number of respondents; M—median; Q_1_—lower quartile; Q_3_—upper quartile; %n_4+5_—percentage of respondents who selected answers ‘agree’ or ‘strongly agree’.

## Data Availability

The data used to support the findings in this study are available from the corresponding author upon request.

## Appendix A

**Table A1:** Learning outcomes realized during the COVID-19 pandemic student volunteering.

Volunteering Activity	Learning Outcomes for Given Faculty							
	Medicine	Dentistry	Pharmacy	Nursing	Midwifery	Laboratory Diagnostics	Physiotherapy	Medical Rescue
initial TRIAGE and questionnaire-based COVID-19 screening of patients	E.U7., G.U2.	E.U8.		D.W1., D.W18.		C.W14., C.U4., C.U8.	A.W19., A.U15.	C.W75., C.U8., C.U58.
interviewing patients and referring to a doctor	D.U5., E.U1., E.U2., E.U38.	D.W4., D.U6.,E.W20., E.U2.,F.U1.	A.W29., A.U21., E.U6., E.U8.	C.W35., C.U2., C.U43., C.U47., D.U15., B.U16. *	C.W8., C.W29., C.W31., C.W32., C.U2., C.U3., C.U40., C.U43.		A.U14., B.W3., C.U1., D.U47.	A.W53., B.W7., C.W24., C.U7.
performing simple medical procedures	E.U28., E.U29., F.U3.–F.U5.	E.U20., F.U5.		C.W7., C.U8., C.U9., C.U15.	C.U7., C.U14.	F.W7.	A.U4., D.U28.	C.U11., C.U50.
working in pandemic emergency departments or hospital wards as temporary residents, ventilator therapy assistants, or nursing assistants	E.U1.–E.U4.,E.U24., E.U38., F.W6., F.W7., F.U3., F.U10., F.U11., G.W11.	E.W2., E.U2., E.U4., F.U1., F.U6., F.U11., G.W34., G.U26.		C.W7., C.W8., C.U2., C.U4., C.U5., C.U26.,C.U45., C.U46., C.U51., D.U9., B.W46. *,B.W47. *,B.U52.–56. *	C.W8., C.W29., C.W30., C.U2., C.U3., C.U40., C.U42., C.U48., D.W58., D.W59., D.W66., D.U47., D.U48.		C.U1., C.U2.,	C.W24., C.W25., C.W56.–61., C.W69., C.W79., C.W80., C.W91., C.U4., C.U7., C.U10., C.U18., C.U19., C.U38.–46., C.U56.
providing patients with advice and information on COVID-19, social distancing, and hospital activity during the pandemic period	C.W13.–C.W15., D.W8., D.W14., D.W15., D.U5., D.U9., G.W3.	C.W3., D.W4., D.W5., D.W11., D.U6.,G.W3., G.W15.	A.W29., A.U21., E.W30., E.U5., E.U6., E.U14., E.U26.	B.W5., B.W13., B.W14., B.U10., B.U11., C.W11., C.W16., B.W22. *, B.U15. *, B.U22.–25. *, B.U39. *	B.W8., B.W18., B.U7., B.U13., C.W14., C.W16.	C.U7.	B.W3., B.W4., B.W12., B.U4., B.U10., D.U47., F.W6., F.U10., F.U18.	B.W7., B.W20., B.W40.
administrative working in a diagnostic laboratory	C.W19., E.W39., E.W40.					D.W9.-D.W13., F.W8., F.U4., F.U8., H.W2., H.W5.–7., H.U1., H.U2.		A.W26., A.U12.
working in a drive-through swab collection unit	C.W19., E.W39., E.U28., F.U3.	F.U5.	A.W18.	C.U9.	C.U7.	A.W19., E.W24., F.W7., F.W8., F.U2., F.U3., H.W3., H.U2.		A.W26., C.W52.
working in sanitary-epidemiological station (e.g., phone duties and conducting epidemiological investigations)	E.W33., G.W3., G.U2.	G.W15.,G.W16., G.U4., G.U19.,G.U21.		D.U4., B.W7.*, B.U5.*		C.W11.		A.U14., B.W31., B.W38., B.U1.
social services (e.g., supporting people with unstable housing, homeless people, and the elderly or immunocompromised citizens)	D.W1., D.W5., D.W6., D.U5., D.U11.	D.W1., D.W4., D.W6., D.W8., D.U6., D.U8.	A.W29., A.W31., A.U19., A.U21.	B.W2., B.U3.	B.W2., B.W8., B.U3., B.U9.		B.W4., B.U10., F.U18.	B.W3., B.W7., B.W20., B.U10.
phone calls to seniors in care facilities and the community to reduce social isolation	D.W5., D.W6., D.U5., D.U11., E.W8., E.W12.	D.W4., D.W6., D.W8., D.U6., D.U8.	A.W29., A.W31., A.U21.,	B.W2., B.W5., B.U3., B.U4., B.U5., D.W12., B.U39. *	B.W2., B.W8., B.U2., B.U3., B.U7., B.U9.		B.W3., B.W4., B.U10., D.U47., F.U18.	B.W7., B.W8., B.W20., B.U10.,
literature reviews on COVID-19 and development of resources for the medical community	B.U10., B.U12., D.W23., D.U17.	D.U13., D.U16.	E.W11., E.W12., F.U2., F.U3.	C.U40., D.U31., C.W6. *,C.W7. *, C.U6. *	B.U18., C.U37., D.U58., C.W6.–8.*, C.U6.-10.*	B.U13., C.W4., E.U27., F.U21., G.U2., G.U3.	E.U2., E.U3.	
development of educational resources for the general public	D.W14.	G.W3., G.U7.	E.W30., E.U26.	C.W11., C.W16.–C.W18., C.U32., B.U22. *, B.U23. *	C.W3., C.W14.–C.W16., C.U26.,	C.W12.	B.W12., B.U4, F.W6., F.W14, F.U10.	B.W29., B.W40.
securing and redistributingpersonal protective equipment and training staff in its use and reuse	C.W20., D.U12., D.U16., F.U3.	C.W5.	A.W20., A.U11., A.U19., C.W31.	B.U11., C.U48., B.U6.*	C.W7., C.W33., C.W34., C.U44., C.U45.	C.U6.	B.U12.	A.W20., A.W25., A.U14., B.W27., B.U1., B.U8., B.U11., C.W51., C.W90.

* Learning outcomes for second-degree (Master’s) studies.Developed on the basis of: Ordinance of the Ministry of Science and Higher Education on the standards of education for the faculties of: medicine, dentistry, pharmacy, nursing, midwifery, laboratory diagnostics, physiotherapy, and medical rescue [28].
